# Assessing the Impact of Intensified Hypertension (HTN) Treatment Guidelines: A Single Center Experience

**DOI:** 10.7759/cureus.32734

**Published:** 2022-12-20

**Authors:** Amir Naqvi, Marc T Zughaib, David Freeman, Tanuj Gupta, Emmanuel Leung, Marcel E Zughaib

**Affiliations:** 1 Cardiology, Ascension Providence Hospital, Southfield, USA; 2 Internal Medicine, Ascension Providence Hospital - Michigan State University College of Human Medicine (MSUCHM), Southfield, USA; 3 Internal Medicine, State University of New York (SUNY) Downstate Medical Center, Brooklyn, USA; 4 Internal Medicine, University of California San Francisco Fresno, Fresno, USA; 5 Heart Institute, Ascension Providence Hospital - Michigan State University College of Human Medicine (MSUCHM), Southfield, USA

**Keywords:** guidelines, management of hypertension, hypertension, cardiology, htn disorders

## Abstract

Introduction: Hypertension (HTN) is an independent risk factor for heart disease, stroke, and premature death. In 2017 there was a shift in the definition of HTN by the American College of Cardiology (ACC), as well as the American Heart Association (ACC/AHA), resulting in lower blood pressure (BP) readings meeting criteria for diagnosis. Our study aimed to explore the impact the change had on a single cardiology practice’s management of patients with HTN.

Methods: We performed a retrospective chart review of a single cardiology practice. We separated the time into two categories: 12 months before and 12 months after the reclassification of HTN categories in November 2017. A paired t-test analysis was done comparing averaged blood pressures (BPs) in each of the two time periods, as well as the number of medications in each time period and several subgroup analyses.

Results: A total of 441 patients were included in the final analysis. Patients were prescribed an average of 2.61 ± 1.20 medications at baseline, and 2.74 ± 1.22 medications post-reclassification (p < 0.0001). There was an average of 0.82 ± 1.28 medication changes per patient. The overall average BP was 133.7 ± 14.1/76.4 ± 9.5 at baseline, and 131.3 ± 13.1/76.7 ± 7.7 after the recategorization [Δ -2.41 (95% CI 1.18-3.63)/0.269 (95% CI -0.29 to 0.459); p<0.0001 for systolic blood pressure (SBP), p=0.467 for diastolic blood pressure (DBP)].

Conclusion: The change in definition of HTN significantly impacted this single cardiology practice. There was a statistically significant increase in antihypertensive medications prescribed with an expected decrease in BP observed in this study.

## Introduction

Hypertension (HTN) is an independent risk factor for heart disease, stroke, and premature death. In 2010, HTN was the leading cause of death and disability-adjusted life years worldwide [1-2]. HTN is defined as a modifiable risk factor, in which lifestyle modifications along with appropriate medications can reduce a person’s blood pressure (BP) as well as decrease the chances of experiencing a cardiovascular event. In the United States, HTN accounted for more cardiovascular disease (CVD) deaths than any other modifiable CVD risk factor and was second only to cigarette smoking as a preventable cause of death for any reason [3].

The American College of Cardiology (ACC), as well as the American Heart Association (AHA), have provided strong evidence-based guidelines to improve cardiovascular health since 1980. In 2017, the ACC and AHA released updated HTN guidelines to replace the Joint National Commission’s (JNC) VIII guidelines. These guidelines recommended new categorization of patients’ BP. This categorization differs from previous recommendations made in the JNC 7 report, with stage 1 HTN now defined as a systolic blood pressure (SBP) of 130-139 or a diastolic blood pressure (DBP) of 80-89 mm Hg, and with stage 2 HTN in the present document defined as SBP greater than 140 mmHg or a DBP greater than 90 mmHg (Table 1) [4].

The severe impact HTN has on end-organ function has been well documented. Unfortunately, effective control of HTN can be difficult to achieve. One study notes 9 out of 10 patients with such diagnoses were receiving pharmacological treatment of their HTN. However, only two out of five of these had optimal BP control [5]. As clinicians move forward with the new ACC/AHA guidelines, it remains to be seen what kind of effect this will have on preventative efforts as well as patient outcomes.

Several studies have noted an increased prevalence of patients meeting the diagnosis of HTN under the updated guidelines [6-8]. However, more evidence is needed to determine if the new guidelines will lead to clinicians changing their management of patients that qualify as HTN. Our study is aimed to investigate whether the recently updated HTN guidelines influenced a single Cardiology practice’s management of patients who meet the newly defined criteria for the diagnosis of HTN.

## Materials and methods

We performed a retrospective chart review of a single cardiology practice. We included patients with essential HTN that presented for an outpatient visit between November 2016 and November 2018. We separated the time into two categories: 12 months before and 12 months after the release of the 2017 ACC-AHA HTN Guidelines in November 2017 (November 15, 2016 to November 15, 2017 and November 15, 2017 to November 15, 2018). All patients were included if they were at least 18 years of age, had essential HTN, and had at least two visits in each of the two time periods (before and after the release of the updated guidelines). Patients were excluded if they did not have at least two visits in either time period.

Blood pressure readings were averaged in each time period. The averages were then compared and overall change relative to guideline release was calculated. In addition to BP readings at each visit, the number and class of each medication were assessed. Furthermore, the numbers of medication changes were included. We accounted for both adjustments in dosage, as well as change in/additional class of medication. 

A paired t-test analysis was done comparing averaged BPs in each of the two time periods, as well as the number of medications in each time period. Subgroup analysis was also done based on whether a patient’s baseline BP was controlled. Further analysis was performed on the basis of number of medication changes that occurred during the post-guideline release time period. Medication changes were considered to be present if there was a dose adjustment, addition or subtraction of a medication class. 

## Results

A total of 898 patients were initially screened. There were 441 patients who met the inclusion criteria and were included in the final analysis. The average age of the patients was 70.4 ± 11.8 years old. 41.7% of the included patients were female. Patients were on an average of 2.61 ± 1.20 medications at baseline, and 2.74 ± 1.22medications post-guidelines (p < 0.0001). A total of 10 patients were not on any BP medications at baseline, while only four patients were not on medications after the guidelines were released. However, after excluding patients who had well controlled BP at baseline (as defined by the new guidelines), only six patients were not on medications at baseline, and all of them were placed on a medication following the release of the guidelines. 

**Figure 1:**
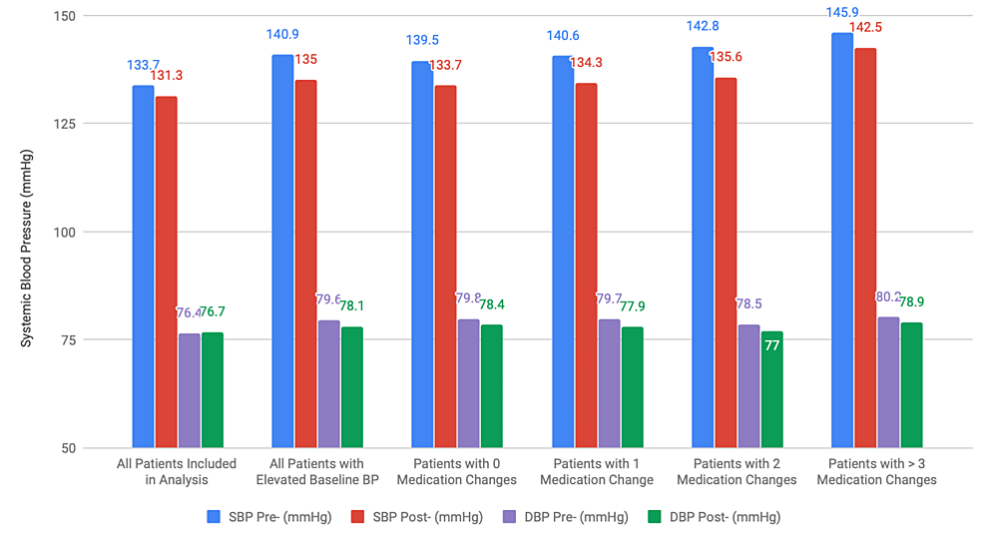
BP trends before and after publication of HTN guidelines. BP, blood pressure; HTN, hypertension

The overall average SBP was 133.7 ± 14.1 at baseline, and 131.3 ± 13.1after the guideline release (p < 0.0001) (Table 1). The overall average DBP was 76.4 ± 9.5 at baseline, and 76.7 ± 7.7 after the guideline release (p=0.467). Patients were on an average of 2.59 ± 0.99 medications in the post-guideline time period. There were an average of 0.96 ± 1.29 medication changes overall in this group. Of these, 0.45 ± 0.83 were due to medication dose adjustment, 0.36 ± 0.59 were due to addition of a new medication class, and 0.20 ± 0.51 were due to removal of a medication class (Figure 1).

**Table 1:** SBP and DBP changes between the pre and post HTN guidelines release. BP, blood pressure; HTN, hypertension; SBP, systolic blood pressure; DBP, diastolic blood pressure

	n	SBP Pre- (mmHg)	SBP Post- (mmHg)	Change in SBP (mmHg)	p-value for SBP	DBP Pre- (mmHg)	DBP Post- (mmHg)	Change in DBP (mmHg)	p-value for DBP
All patients included in analysis	441	133.7	131.3	-2.41	p<0.0001	76.4	76.7	0.269	p=0.467
All patients with elevated baseline BP	279	140.9	135	-5.88	p<0.0001	79.6	78.1	-1.5	p=0.0010

 

There was an average of 0.82 ± 1.28 medication added to each patient's regimen. The largest medication class represented was beta-blockers, followed by angiotensin converting enzyme inhibitors (ACEi), thiazide diuretics, and angiotensin receptor blockers (ARBs). Patients were on an average of 2.59 ± 0.99 medications in the post-guideline time period. There were an average of 0.96 ± 1.29 medication changes overall in this group. Of these, 0.45 ± 0.83 were due to medication dose adjustment, 0.36 ± 0.59 were due to addition of a new medication class, and 0.20 ± 0.51 were due to removal of a medication class.

There were 128 (45.9%) of the above 279 patients who did not have any medications changed throughout the study time period. They had an average blood pressure of 139.5 ± 12.3/79.8 ± 8.45 at baseline pre-guidelines and 133.7±11.7/78.4±7.45 in the post-guideline period (Δ -5.78 (95% CI 3.70-7.86)/-1.33 (95% CI 0.08-2.58); p<0.0001 for systolic blood pressure and p = 0.0366 for diastolic blood pressure) (Table 2). 

**Table 2:** BPs in patients that had 0, 1, 2, or 3 BP medications changed during the pre and post HTN guideline release. BP, blood pressure; HTN, hypertension; SBP, systolic blood pressure; DBP, diastolic blood pressure

	n	SBP Pre- (mmHg)	SBP Post- (mmHg)	Change in SBP (mmHg)	p-value for SBP	DBP Pre- (mmHg)	DBP Post- (mmHg)	Change in DBP (mmHg)	p-value for DBP
Patients with 0 medication change	128	139.5	133.7	-5.78	p<0.0001	79.8	78.4	-1.33	p=0.0366
Patients with 1 medication change	90	140.6	134.3	-6.25	p<0.0001	79.7	77.9	-1.8	p=0.021
Patients with 2 medication changes	34	142.8	135.6	-7.24	p=0.0146	78.5	77	-1.47	p=0.31
Patients with > 3 medication changes	27	145.9	142.5	-3.43	p=0.35	80.2	78.9	-1.34	p=0.48

.Of the 279 patients with elevated BP at baseline, 90 (32.3%) had one medication change during the study period. The average BP of this group was 140.6 ± 10.9/79.7 ± 9.76 at baseline and 134.3 ± 11.9/77.9 ± 8.43 in the post-guideline period [Δ -6.25 (95% CI 3.93-8.57)/-1.80 (95% CI 0.28-3.31); p<0.0001 for SBP and p = 0.021 for DBP]. By the end of the study period, patients were taking an average of 2.69 ± 1.23 medications each (Table 2).

Thirty-four (12.2%) of the 279 patients with elevated BP at baseline had two medication changes during the study period. The average BP of this group was 142.8 ± 11.3/78.5 ± 10.7 at baseline and 135.6 ± 13.5/77.0 ± 7.13 in the post-guideline period [Δ -7.24 (95% CI 1.53-12.95)/-1.47 (95% CI -1.43-4.38); p=0.0146 for SBP and p = 0.31 for DBP, significant for SBP but not DBP change]. After one year of the guidelines being released, patients were on an average of 2.62 ± 1.28 medications (Table 2).

There were 27 (9.7%) of the 279 above patients with at least three and as many as seven medication changes during the follow-up period. The average BP of this group was 145.9 ± 13.8/80.2 ± 9.28 at baseline and 142.5 ± 15.2/78.9 ± 10.1 in the post-guideline period [Δ -3.43 (95% CI -3.91 to 10.77)/-1.34 (95% CI -2.46 to 5.16); p=0.35 for SBP and p = 0.48 for DBP, neither statistically significant]. After one year post-guideline release, patients were taking an average of 3.63 ± 1.04 medications each. When comparing this to the group that had no medication changes, this group did have significantly greater number of medications with p<0.0001. This was also true when comparing against the group with one or two medication changes (Table 2).

## Discussion

The 2017 ACC-AHA guidelines for management of HTN recommend a goal BP of less than 130/80 for the vast majority of hypertensive patients [4]. This is largely based on data from the SPRINT trial, which found a decrease in all cause mortality, as well as a decrease in fatal and nonfatal cardiovascular events in patients with a target systolic blood pressure of less than 120 mmHg [9]. The methods toward which this goal was achieved were left to the provider’s discretion.

Our study demonstrated that the guidelines were successfully implemented, with a statistically significant, albeit modest reduction in SBP when considering the aggregate of patients screened. This was likely predominantly driven by the patients with elevated BP at baseline (in the pre-guideline time period). There was also a statistically significant increase in medications prescribed from the pre-guideline to the post-guideline release time frames. A number of the patients included in this analysis had relatively well-controlled BP at baseline, with SBP less than 130 mmHg and DBP less than 80 mmHg. As a result, we performed a subgroup analysis of all of the patients who had BP above these parameters to assess the impact on their BP control. This revealed a much more meaningful decrease in both SBP and DBP as compared with the overall aggregate group of patients. 

 In addition to the emphasis on medication management, the updated ACC-AHA HTN guidelines also stressed the importance of lifestyle changes in BP control. Our study indirectly confirms the impact this can make on improving SBP. This is highlighted by the fact that 128 patients had no medication changes, yet they still had a statistically significant decrease in both SBP and DBP. 

 We did find that with each additional medication change, there was a trend for a greater effect seen on BP control. This was true only up until three medication changes were done, however. There are several possibilities as to why this was the case. Firstly, the group with greater than three medication changes was limited by a small number of patients and may not carry enough statistical weight to show a true correlation. Another possibility is that these patients had a greater level of resistant HTN. This is supported by the fact that they were on a significantly greater number of medications (3.63 medications on average) and yet still had poorer BP control. Yet another potential explanation is that there may be a greater degree of medication non-adherence in this group. 

 For the overall population of patients screened, as well as the groups with two medications changed or greater than three medications changed, DBP improvement was not statistically significant. As previously mentioned, the study population was relatively older (70.4 + 11.8 years old) and is thus more likely to have isolated systolic HTN. This was indeed the case in our study population, with every subgroup having mildly elevated SBP, while DBP was at target. The pathophysiology behind this is related to loss of arterial wall compliance and stiffening of arterial walls with age. This in turn leads to increased cardiovascular risk [10].

 One interesting finding was the predilection for beta-blockers over other antihypertensive agents. This can likely be explained by the fact that the population studied was from a cardiology practice and thus has a much higher likelihood of comorbidities such as atherosclerotic CVD or heart failure, and thus an indication for beta-blockers over other medications.

 Our study is limited by the fact that it was done in a retrospective fashion, as well as by the relatively small numbers in the subgroup analyses. Another limitation is the fact that this is based on a single cardiology practice, which may reduce generalizability. 

## Conclusions

The 2017 ACC-AHA guidelines for management of HTN did significantly impact this single cardiology practice evidenced by a significant reduction in BP. There were increases in medications prescribed in order to meet the standards set by the new HTN guidelines.
